# Deep Sequencing and Molecular Characterisation of BK Virus and JC Virus WHO International Reference Materials for Clinical Diagnostic Use

**DOI:** 10.3390/v15061289

**Published:** 2023-05-30

**Authors:** Sheila Govind, Martin Fritzsche, Adrian Jenkins, Megan H. Cleveland, Peter M. Vallone, Neil Almond, Clare Morris, Neil Berry

**Affiliations:** 1 Division of Infectious Disease Diagnostics, National Institute for Biological Standards and Control (NIBSC), South Mimms EN6 3QG, UK; 2 Division of Analytical and Biological Sciences, National Institute for Biological Standards and Control (NIBSC), Medicines and Healthcare Product Regulatory Agency (MHRA), South Mimms EN6 3QG, UK; 3 Applied Genetics Group, National Institute of Standards and Technology, Gaithersburg, MD 20899, USA

**Keywords:** polyomaviruses, International Standard, deep sequencing, genetic polymorphisms, commutability, clinical diagnostics

## Abstract

Background: Reactivation of JC and BK polyomaviruses during immunosuppression can lead to adverse clinical outcomes. In renal transplant recipients, BKV-associated nephropathy can result in graft loss, while in patients with autoimmune disorders, prolonged immunomodulatory drug use can cause rare onset of progressive multifocal leukoencephalopathy due to JCV reactivation. In such patients, accurate BK and JC viral load determinations by molecular technologies are important for diagnosis and clinical management; however, comparability across centres requires effective standardisation of diagnostic molecular detection systems. In October 2015, the WHO Expert Committee for Biological Standardisation (ECBS) established the 1st WHO International Standards (ISs) for use as primary-order calibrants for BKV and JCV nucleic acid detection. Two multi-centre collaborative studies confirmed their utility in harmonising agreement across the wide range of BKV and JCV assays, respectively. Previous Illumina-based deep sequence analysis of these standards, however, identified deletions in different regions, including the large T-antigen coding region. Hence, further detailed characterization was warranted. Methods: Comprehensive sequence characterisation of each preparation using short- and long-read next-generation sequencing technologies was performed with additional corroborative independent digital PCR (dPCR) determinations. Potential error rates associated with long-read sequencing were minimised by applying rolling circle amplification (RCA) protocols for viral DNA (circular dsDNA), generating a full validation of sequence identity and composition and delineating the integrity of full-length BK and JC genomes. Results: The analysed genomes displayed subpopulations frequently characterised by complex gene re-arrangements, duplications and deletions. Conclusions: Despite the recognition of such polymorphisms using high-resolution sequencing methodologies, the ability of these reference materials to act to enhance assay harmonisation did not appear significantly impacted, based on data generated by the 2015 WHO collaborative studies, but highlights cautionary aspects of IS generation and commutability for clinical molecular diagnostic application.

## 1. Introduction

The two most clinically relevant polyomaviruses associated with human pathologies are the BK virus (BKV or BKPyV) and JC virus (JCV or JCPyV) [1]. Primary infection of either virus in early childhood is typically asymptomatic and population-prevalent [2]. In immunocompromised patients, however, reactivation in renal transplant recipients, for example, can lead to BKV-associated nephropathy which can result in serious complications and graft loss [3] and is associated with renal failure in hematopoietic stem cell transplantation HSCT patients [4]. Prior JCV infection can be reactivated through immunomodulatory drug treatment, leading to the onset of progressive multifocal leukoencephalopathy (PML), a fatal demyelinating disease caused by the lytic infection of oligodendrocytes by JCV [5].

Viral load measurements by molecular assays are a key component in the clinical diagnostic pipeline of these immunocompromised patient groups, required to guide clinical management decisions, when serum BKV load exceeds recommended thresholds [6,7]. However, inevitable inter-assay variability can lead to potentially wide discrepancies between viral load estimates across laboratories, which obfuscates attempts at diagnostic comparability and in doing so compromises equivalence of therapeutic decision points and intervention [8,9].

Assay standardisation is hence vital for accurate molecular diagnosis and consistent treatment decisions between operators, care settings and countries [10]. To facilitate this, two World Health Organisation (WHO) International Standards (ISs), one for BKV (NIBSC, South Mimms, UK, catalogue #14/212 refs [11,12]) and the other for JCV (NIBSC, South Mimms, catalogue #14/114 [13]) were established in conjunction with the WHO Expert Committee for Biological Standardisation (ECBS) in 2015. As a prerequisite to establishment, each candidate reference preparation was independently evaluated by multiple expert laboratories worldwide using frontline diagnostic assays. Each preparation comprised high-titre cell-culture-propagated virus achievable only through passage of viral stocks. For the JCV WHO IS material, the viral stock was previously genotyped by Dr. Alexis Dumoulin and Dr. Hans Hirsch (personal communication by donating laboratory) and classified as genotype 1 subtype A, the original material propagated in Cynomolgus macaque kidney cells. The material for the BKV WHO ISs was genotype 1 subgroup Ib-2, propagated using human foetal lung fibroblast cell line MRC-5. These two selected candidate preparations proved to be most effective at harmonising data when used as primary calibrants, evaluated as part of an International collaborative study, reducing measurement variability by several orders of magnitude, demonstrating their suitability for assay harmonisation, and subsequently approved by the WHO ECBS. Post-approval, these WHO-ECBS-endorsed reference preparations were made available to the scientific community as primary biological standards for calibration of both commercially available IVD molecular assays as well as in-house assays frequently used in clinical settings.

In 2017, two reports detailed the measurement of the BKV and JCV WHO ISs, using digital droplet PCR (dPCR) across multiple genomic regions and Illumina next-generation sequencing [14,15], raising a potential under quantification when using dPCR assays targeting the large T-antigen (LTAg) region, which was corroborated by lower short-read coverage across this region. To further address this phenomenon and complement our initial analyses undertaken using Sanger technology, we assured fitness for purpose of each WHO IS, using genome amplification and different NGS approaches extending to long-read (Nanopore) analyses, incorporating analytical methods anticipated to be employed by clinical scientists in diagnostic pipelines. This analysis generates a more thorough evaluation of the genome sequence and context, including descriptions of variations and quasi-species structure which are important additional data for these nucleic acid amplification testing (NAT) materials. 

The quality control and bioinformatic analyses employed enabled derivation of a hybrid de novo analysis complemented by standard localised analyses. We identified quasi-species (low-frequency non-reference mutations) and major structural populations within the two IS preparations. The impact of these genomic re-arrangement features on viral copy number determination was further assessed using dPCR. The implications of these findings for use of the WHO ISs for assay calibration are discussed in context of the provision of reference materials for use in a clinical setting.

## 2. Materials and Methods

### 2.1. Nucleic Acid Extraction for Sequencing

Vials of lyophilised WHO IS materials (BKV 14/212) and (JCV 14/114) were removed from −20 °C storage and hydrated in 1 mL of molecular-grade water according to the instructions for use provided. Samples were manually extracted using QIAamp DNA mini kit according to the manufacturer’s instructions (QIAGEN, Hilden, Germany). For next-generation sequencing DNA extracts from multiple vials were pooled and purified with Agencourt^®^ AMPure^®^ XP magnetic beads (Beckman Coulter, Los Angeles, CA, USA) and concentrated according to the manufacturer’s instructions. The purity and integrity of DNA extracts were confirmed using the Qubit fluorometer using the Qubit dsDNA HS Kit (Thermo Fisher, Boston, MA, USA).

### 2.2. Sanger Sequencing

For BKV, Sanger sequencing PCR amplicons were generated to cover the entire genome as previously described [16] and sequenced on a 3130 XL Genetic Analyser (Applied Biosystems, Boston, MA, USA) using conventional methodologies. Sequencing data were assembled and analysed using Geneious v. 10.2 software [16]. For JCV, the full genome sequencing of the donated isolate was not completed using Sanger methods, but genotyping was verified as described in [13]. 

### 2.3. Nucleic Acid Extraction for Digital PCR

Each vial of WHO ISs was reconstituted in 1 mL of molecular biology grade water. DNA extractions were performed with the EZ1 Virus Mini Kit 2.0 on the EZ1 Advanced XL system (QIAGEN, Hilden, Germany) following the manufacturer’s recommended procedures. A total of 200 µL of hydrated material was extracted and eluted into 90 µL. For each material, five replicate extractions were performed and then pooled.

### 2.4. Droplet Digital PCR (dPCR)

Each extracted material was diluted 1:10 into molecular biology grade water before dPCR reactions were performed in triplicate. Each reaction contained 1× ddPCR Supermix for Probes with dUTP (Bio-Rad Laboratories, San Francisco, CA, USA), 312.5 nM of each primer (Eurofins, Luxembourg) and FAM/BHQ Plus probe (LGC, Biosearch Technologies, Hoddesdon, UK) and 2 µL of diluted template DNA in a total reaction volume of 22 µL. Assays were designed in-house [17]. Emulsion droplets were generated on the AutoDG instrument (Bio-Rad Laboratories, San Francisco, CA, USA) according to the manufacturer’s recommended procedure then amplified on the ProFlex 9 (Thermo Fisher, MA, USA) thermal cycler using the following program: initial denaturing at 95 °C for 10 min, then 60 cycles of (94 °C for 30 s and 60 °C for 60 s (with ramp rate of 2.5 °C per second)), then a final cycle of 98 °C for 10 min and a hold at 4 °C at the end of the program. Droplets were read on the QX200 Droplet Reader (Bio-Rad Laboratories, CA, USA) according to the manufacturer’s recommended procedure with detection in the FAM channel. Primer and probe sequences and locations for BKV and JCV dPCR measurements are shown in Table 1.

### 2.5. Data Analysis for Droplet Digital PCR

Thresholds separating negative and positive droplets were drawn manually in the QuantaSoft Software, version 1.7.4.0917. Data exported to Microsoft Excel were analysed using a droplet volume of 0.7584 nL. Lamba (λ) values were calculated using the formula λ = −ln (fraction of negative droplets). The lambda value was adjusted for the extraction concentration and dilution into the PCR reaction. This value was then divided by the droplet volume, resulting in copies/mL. 

### 2.6. Short-Read Sequencing

Dual-indexed, pooled sequencing libraries were generated using the Nextera XT DNA library preparation kit (Illumina, FC-131-1096). Libraries were sequenced paired-end on the MiSeq platform (Illumina, SY-410-1003) for 2× 250 cycles, using v2 reagents (Illumina, MS-102-2003) and the “FASTQ only” workflow. Initial demultiplexing was performed onboard by the MiSeq Reporter software. Raw sequencing reads were adapter and quality trimmed by Trim Galore v0.4.4 for a minimum Phred score of Q30 and minimal read length of 75 bp. Trimmed reads were aligned against BKV (JN192431.1) and JCV (J02226.1) reference sequences using BWA v0.7.17 and de-duplicated using Picard v2.18.12. Read coverage calculations, variant calling and consensus sequence generation were performed using Samtools v 1.9.

### 2.7. Long-Read Sequencing

To generate sufficient input material for long-read sequencing and to reduce high error rates associated with this technology by generating redundant sequences, viral DNA (circular dsDNA) was amplified via rolling circle amplification (RCA). A detailed protocol was published by Stevens et al. [18]. Briefly, the Illustra TempliPhi Amplification 100 Kit (GE Healthcare, Chicago, IL, USA), which contains phi29 DNA polymerase and random hexamer primers, was used to create individual linear dsDNA molecules that contain concatemeric whole-genome units of virus. After 1× AMPure (Beckman Coulter, Brea, CA, USA) purification, rolling-circle amplified DNA was used for 1D Nanopore ligation library preparation (Oxford Nanopore Technologies, Oxford, UK). Samples were sequenced individually on the MinION sequencer, using R9.4 and R9.5 flow cells (Oxford Nanopore Technologies, FLO-MIN106/FLO-Min107). MinION reads were base called using albacore v2.3.3, adapter trimmed with Porechop v0.2.2 and quality trimmed by BBduk for a minimum Phred score of Q9 and minimal read length of 15,000 bp. Read-segmentation, dot-plot generation and sub-read clustering was performed using custom R scripts.

## 3. Results

### 3.1. Sanger Sequence Analysis

PCR amplified products consisting of 500–1000 bp fragments of BKV and JCV across the respective genomes were assembled using Geneious software and aligned with reference sequence JN192431.1 and J02226.1, respectively. Due to incomplete sequence data generated by this approach, and specifically discrepancies in the LTAg region, the main focus of further sequence analysis related next generation sequencing approaches. 

### 3.2. Short-Read Sequencing

**Figure 1:**
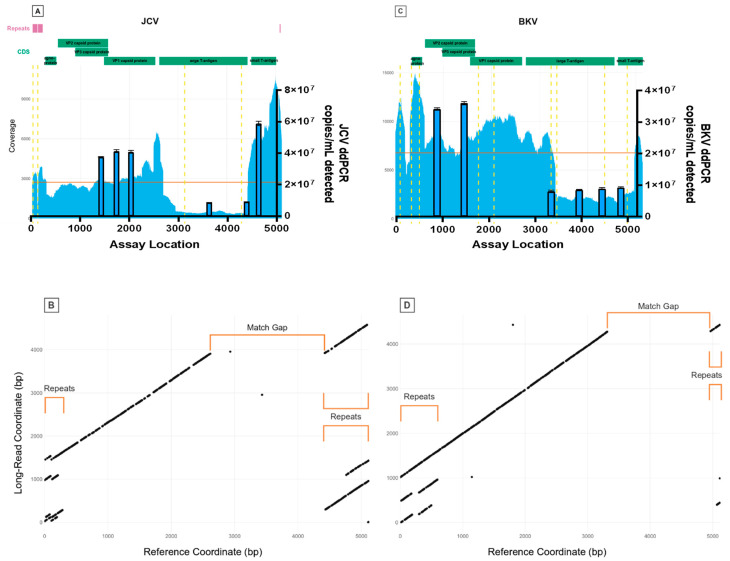
Overview of short- and long-read NGS analysis of JCV and BKV ISs (**A**). Absolute sequencing coverage across JCV reference genomes. Horizontal lines indicate mean coverage, dashed vertical lines indicate SNP positions (>1% minor allele frequency). The dPCR data depicted as blue bars in dPCR copies correspond with NGS coverage data. (**B**). Dot plot of JCV reference genomes (*x*-axis) plotted against one representative sub-read, generated via Nanopore sequencing (*y*-axis). Regions of no match and repetitive regions correlate with regions of low and high sequencing coverage. (**C**) and (**D**) show respective comparable analyses for BKV as a direct comparison with JCV for absolute sequence coverage and dot-plot analyses, respectively.

**Figure 2:**
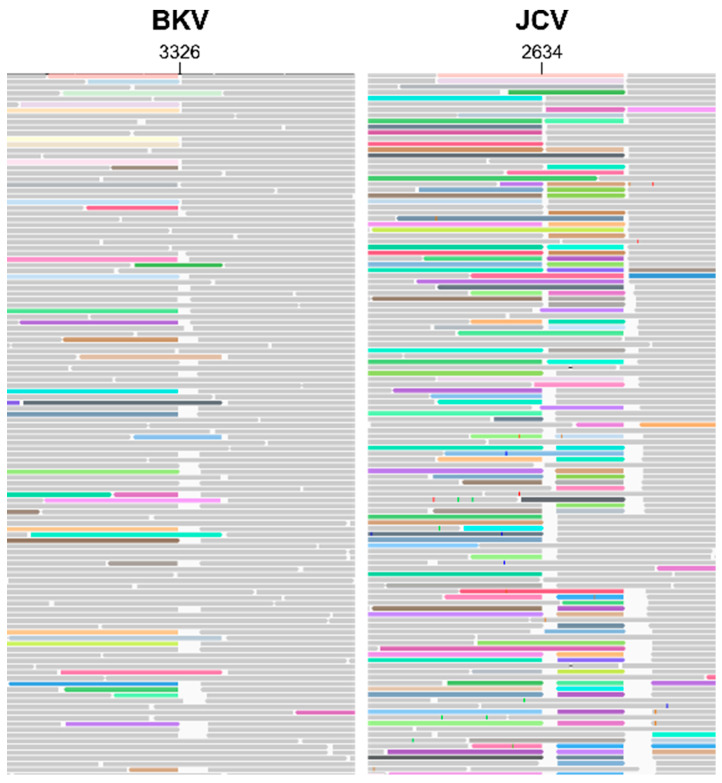
Alignment tracks of BKV and JCV ISs. BKV IS and JCV IS alignment tracks around nucleotide position 3326/2634, respectively, show increased read clipping. Soft- and hard-clipped reads are highlighted.

Firstly, we analysed the WHO ISs for BKV and JCV using amplicon-based Illumina deep sequencing. A total of 257,492 and 93,195 reads were aligned to BKV reference JN192431.1 and JCV reference J02226.1, respectively, detecting nine high-confidence (minor allele frequency > 1%) Single nucleotide polymorphisms (SNPs) for BKV and four for JCV (Table 2). For BKV, two mutations result in an amino acid change, and one results in a premature stop codon; for JCV, two mutations result in an amino acid change. For both viruses, read mappings displayed abrupt variations in sequencing depth (Figure 1A,C) with several locations of increased read-clipping (Figure 2) and junctional reads. Together, these observations suggest presence of viral subpopulations containing diverse structural variants (large-scale insertions, deletions, or other re-arrangements). Junctions for suspected structural variants were detected within BKV at the following positions: 203, 301, 610, 3326, 4966 and 5106; and within JCV at the following positions: 108, 128, 212, 297, 2493, 2634, 2693, 2945, 4423 and 4872.

One central limitation of short-read NGS is the difficulty of resolving structurally complex genomes containing repeats or other large-scale re-arrangements. Without some form of long-range information, it is impossible to distinguish true variation from mapping artefacts and to phase distant variants within the genome. Hence, to overcome these limitations, we employed Nanopore long-read sequencing to further characterise the putative structural variants present in the BKV and JCV IS.

### 3.3. Long-Read Sequencing

**Figure 3:**
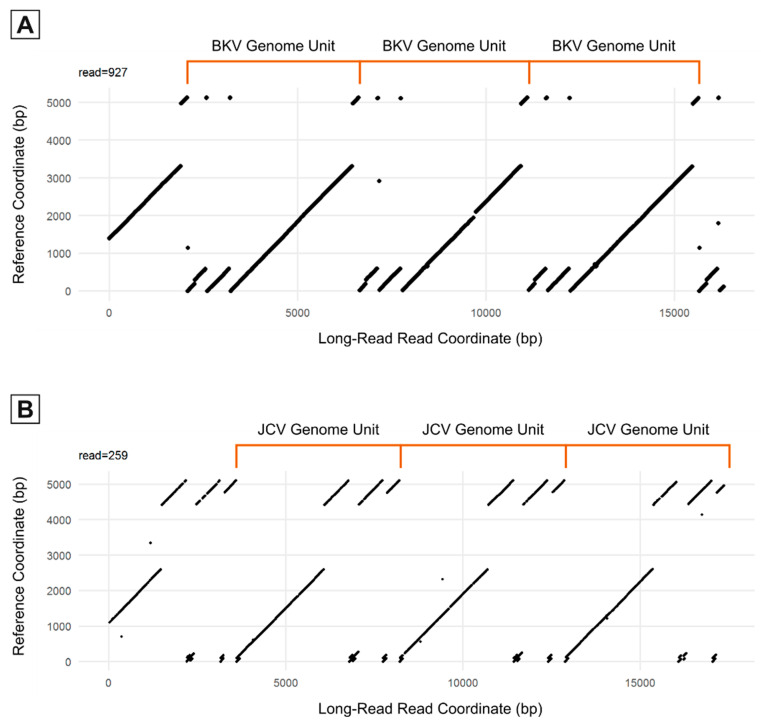
Representative Nanopore reads containing concatemeric copies of the full BKV IS and JCV IS genomes. Reference genomes (*y*-axis) of BKV (**A**) and JCV (**B**) are plotted against one representative Nanopore read (*y*-axis). Each read contains 3 full copies of BKV or JCV.

**Figure 4:**
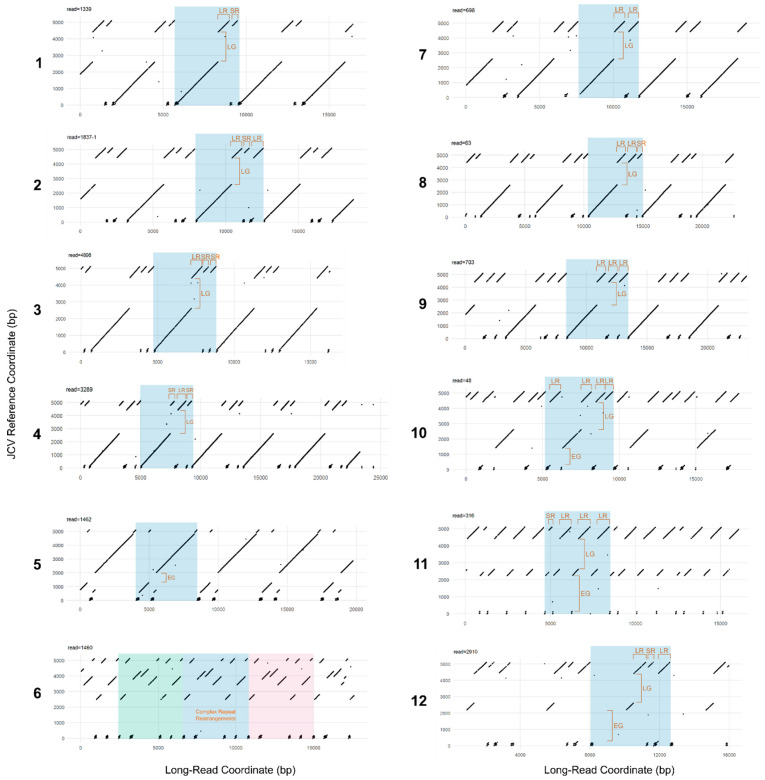
JCV structural variant quasi-species. JCV reference genomes (*y*-axis) are plotted against one representative Nanopore read (*x*-axis) belonging to a quasi-species with a specific set of genomic re-arrangements. Individual genomic units and characteristic match gaps and repeats are highlighted. Reads have been assigned to groups of quasi-species based on the similarity of the dot plot. Estimated frequencies of identified quasi-species: (**1**) 1.0%, (**2**) 5.1%, (**3**) 1.0%, (**4**) 1.0%, (**5**) 8.2%, (**6**) 1.0%, (**7**) 15.3%, (**8**) 31.6 %, (**9**) 1.0%, (**10**) 1.0%, (**11**) 32.7%, (**12**) 1.0%. LR: long repeat; SR: short repeat; LG: late gap; EG: early gap.

Following rolling circle amplification (RCA) of each viral reference genome to generate concatemeric genomic units, dot-plot analysis of whole Nanopore reads containing repeats of the BKV and JCV genomes (Figure 3) revealed large-scale deviations of individual viral genome copies from their respective references. Both regions of sequence duplication and interruptions of matched sequences correlate closely with abrupt shifts in sequencing depths (Figure 1B,D), further indicating structural re-arrangements in both BKV and JCV. Manual analysis of individual long reads revealed the nature of these re-arrangements and their positional relation to each other. A detailed overview of representative JCV structural variant quasi-species is shown in Figure 4. While BKV displayed a similar spectrum of re-arrangements, the number of reads with a sufficient coverage of the total genome was too low for a similar, semi-quantitative analysis. For both BKV and JCV, the majority of viral genomes possessed either a partial or total deletion of the large T-antigen region (indicated by gaps in the diagonal line of the dot plots). These regions yielded correspondently low coverage of short reads.

In addition, both virus sequences display complex patterns of self-similarity indicated by parallel lines in the dot plots, where the distance between the lines is equal to the distance between the repeats. In the case of BKV, the 5′ region shows two tandem repetitions of the first 610 bp, with approximately 50% of repeats exhibiting a deleted region between 203 and 301 bp (exact coordinates differ among individual genomes). The repeats encompass the origin of replication, the coding sequence of the agnoprotein (376–576 bp) and small parts (~20 bp) of terminal 3′ region of the genome, which are interspersed between the repeat units. The coding sequences of the three capsid proteins VP1, VP2 and VP3 are not altered in the majority of analysed genomes. However, it is noteworthy that the major allele SNP 1728 A/T introduces a premature stop codon, potentially inactivating VP1. 

JCV quasi-species displayed a great variety of structural re-arrangements (Figure 4). The key features observed were as with BKV, JCV genomes show a complex expansion of the 5′ region; an extreme example of this phenomenon is shown in Figure 4 (part 6). The 196 bp tandem repeat region (containing two repeat units) appears partially duplicated and interspersed with a ~700 bp region from the 3′ terminal end that encompasses the intron and coding sequence of the small T-antigen and part of the origin of replication (“Long Repeat” in Figure 4). The interspersed regions appeared in various degrees of truncation (“Short Repeat” in Figure 4) and duplication. The duplicated 3′ genome regions correspond well to 3′ peaks in the coverage plot. Similar to BKV, the large T-antigen coding sequence is deleted in the majority of analysed JCV genomes (“Late Gap” in Figure 4). In addition, deletions of the agnoprotein and all three capsid protein coding sequences could be identified in individual genome copies (“Early Gap” in Figure 4). In the most prevalent quasi species (Figure 4, part 11), both the capsid proteins and the large T-antigen were deleted. Only a very small region between ~2493 and 2634 bp appeared to be intact in virtually all analysed genome copies. This corresponds to the intergenic region between VP1 and large T-antigen. Importantly, no genome copy that contained the full JCV reference sequence was detected. Nevertheless, despite the complex re-arrangements, duplications and deletions, the overall length of individual genome units remained at ~4900 bp suggesting some form of physical constraint on genome length variation.

### 3.4. Digital PCR

Concurring with the sequencing results, digital PCR (dPCR) assays showed significant populations with deletions in the large T-antigen regions of both BKV and JCV (Table 3). Additionally, dPCR supported repeats in at least one population in the small T-antigen region of JCV (assay JC-M). For JCV, the assays in the T-antigen region (JC-K, JC-L) quantified genome copies to be 20% of the assays amplified in the VP regions (JC-H, JC-I, JC-J). For BKV, the assays in the T-antigen region (BK-D, BK-E, BK-F) yielded concentrations of about 25% of the assays in the VP regions (BK-A, BK-B).

## 4. Discussion

Detailed characterisation of the genetic composition of candidate or established reference materials for NAT assessments is now a crucial part of any workflow when evaluating their provenance and purpose suitability. It is important to recognise, however, that such analyses should remain part of an overall picture encompassing many performance indicators where the early generation of such materials, even if not ideal, can facilitate significant steps towards data harmonisation and assay validation. In this report we provide a comprehensive analysis of the sequence composition of both JC and BK WHO IS reference materials through a combination of short and long read deep sequencing approaches to provide complementary analyses of their viral sequence heterogeneity. Both long and short read deep sequencing approaches identified viral subpopulations and complex structural variants within both WHO IS preparations including the presence of major deletions and some duplications. 

These findings were further supported by digital PCR analysis, providing observations which both corroborate and extend recent findings described by Bateman et al. [15] and Greninger et al. [14]. Factors determining the generation of major deletions, such as in the LTAg region, following viral propagation in vitro requires further investigation given the paucity of suitable clinical material and the tendency to rely on in vitro-propagated virus stocks. Indeed, the biological processes of this phenomenon warrant further study, both as in vitro culture-derived artefacts and/or frequency in clinically-derived materials. 

Moreover, the impact of such genome re-arrangements on copy number estimation using dPCR techniques to determine copy number variation across the BKV IS genome indicates the complementary value of this approach to augment sequencing-based approaches to investigate material integrity. These investigations clearly demonstrate variation in copy number are dependent on the amplification target region, for example, when comparing LTAg and the VP2 regions. Moreover, understanding where genome re-arrangements are most likely to occur, and the circumstances in how they might arise, ultimately informs assay design, as these regions would seem to be probably best avoided as diagnostic amplicon targets. In regard of this particular reagent, calibration of secondary reference materials to the WHO ISs would be better performed outside of the LTAg region. Use of noncoding regions may provide valid alternatives which are functionally more conserved with respect to replicative biology, as has been demonstrated for these polyomaviruses, HIV and HCV. However, it is also true that the more variable nature of the noncoding regions of both JC and BK polyomaviruses in human samples and cell-culture-derived products indicates that their use as target regions could strongly bias the results. Hence, sequencing of secondary reference materials should be performed in order to verify these noncoding regions are intact. 

Despite these observations, data from evaluations performed by expert laboratories in collaborative studies support the use of the BKV and JCV WHO IS materials towards improving the agreement of molecular assays across laboratories. Notably, data presented in the BKV study demonstrate that even an imperfect whole virus-based reference harmonised participant data far better than a sequence-perfect plasmid standard [19], further acknowledging the potential pitfalls of determinations based on artificial plasmid-based calibrants compared to reference materials that require both extraction and amplification. Context provided by studies addressing commutability issues are therefore an important consideration when preparing, evaluating and implementing candidate or established reference materials as calibrants [20,21]. Caution is particularly needed with regards to recommendations that synthetic nucleic acid materials may provide superior standards to calibrate clinical molecular diagnostics assays where naked DNA-derived materials will by definition be either partly or completely excluded from an extraction protocol designed for more complex clinical materials. Commutability issues therefore require continuous evaluation and interpretation with all aspects deserving of scrutiny and revision as more information becomes available over time. 

Hence, this study further highlights a recurring theme when developing reference materials for molecular diagnostic assays, in that no type of prepared viral material will ever likely perfectly reflect the biological reality of the patient sample. Whilst it may seem preferable to employ clinical material to create a calibrant to more directly address commutability issues, this also has its drawbacks. Viral sequence heterogeneity in any particular clinical sample may be representative of the viral populations in that individual patient but not necessarily in other patients, which may reflect a different population of viruses in wider circulation. Alternatively, the use of established virus strains propagated in cell lines may also be problematic both in terms of how representative a quasi-stable cell line is to real-life clinical materials and precisely how stable the relationship is between the initial isolate and cell line, understanding that changes may occur during in vitro propagation. The latter consideration may actually be the more manageable and perhaps preferable to the use of synthetic constructs. Acceptance of quantitative BK PCR assays calibrated against the WHO IS for BK virus, allowing reporting in IU, will further enable better support of transplant patients allowing laboratories to make meaningful comparisons between measurements of BKV DNA levels [21,22].

However, whatever system is used for calibration the potential impact of the gene re-arrangements found in both calibrants described here and in clinical samples on viral estimations must be factored in. Detailed analyses using short and long read sequencing in the wider context of assay performance will further establish the genetic integrity and utility of materials designed to facilitate molecular nucleic acid measurement in clinical diagnosis. In doing so, this will ensure that assays with sufficient accuracy are employed to improve clinical management of patients and become more widely established.

## Figures and Tables

**Table 1:** Primer and Probe locations for the BKV and JCV dPCR assays. Start/End base pair locations for BKV and JCV are given relative to accession numbers JQ713822.1 and J02226.1, respectively.

Assay	Forward Primer	Reverse Primer	Probe(5′ FAM/3′ BHQ-1 Plus)	Region Targeted	Start	End	Amplicon Size
BK-A	tgctgggtttgctgctttaattc	gccatgcctgattgctgatag	ttgggatcacaaagtttcca	VP2	812	940	129
BK-B	ccaggaggtgctaatcaaagaac	gcaggtgttacagtcccgta	ctcctcaatggatgttgcc	VP2	1422	1501	80
BK-D	ccatgtcctgaaggcaaatcct	ggctaacctttgagctaggtgta	ttgattcagctcctgtcc	Large T Antigen	3290	3392	103
BK-E	ctcacactttgtctctactgcatac	aacccagaagagcctgaagaaac	ttaatttccaagacacctgctt	Large T Antigen	3910	3987	78
BK-F	ggtggtgttgagtgttgagaatc	ggagtcctggtggagttccttt	tgctgttgcttcttcatca	Large T Antigen	4382	4495	114
BK-G	aggccattccttgcagtacag	cctggagtagctcagaggtttg	tctgggcaaagaggaaaatcagc	Large T Antigen	4812	4882	71
JC-H	caccaggaggtgcaaatcaaag	gggccatcttcatatgcttcaagag	tctgctcctcaatggatgttg	VP2/VP3	1370	1475	106
JC-I	ggacatgcttccttgttacagtg	cacagcctcccacatgagta	ttccactacccaatctaaatgag	VP1	1693	1786	94
JC-J	tgccacagtgcaatctcaag	ggaacccaacattcaacaggata	atgaacacagagcacaaggcgta	VP1	1990	2076	87
JC-K	ctggtcatgtggatgctgtcaa	gccagcaggctgttgatac	ccctttgtttggctgctaca	Large T Antigen	3597	3657	61
JC-L	caggtcttcatcccacttctcatta	ggtgccaacctatggaacagat	tattccaccaggattccca	Large T Antigen	4355	4427	73
JC-M	cccagcaatgaagagcttcttg	tgcaaggaatggcctaactg	taagtcacacccaaaccattg	Small T Antigen	4539	4731	193

**Table 2:** Nucleotide sequence variation across the WHO BKV IS (14/212) and WHO JCV IS (14/114) genomes compared with reference sequences. Only SNPs above 1% minor allele frequency are reported.

Nucleotide Position	Reference Allele	Variant Allele	Variant Allele Frequency	Amino Acid Change	Affected Gene
*BKV (JN192431.1)*					
102	A	C	0.94	NA	NA
337	G	A	1	NA	NA
502	G	A	0.18	Glu/Lys	Agnoprotein
1728	A	T	1	Arg/STOP	VP1
2050	A	C	1	Asn/Thr	VP1
3238	T	G	1	Thr/Thr	Large T-Antigen
3358	A	T	1	Thr/Thr	Large T-Antigen
4351	G	A	1	Ser/Ser	Large T-Antigen
4818	G	A	1	His/His	Small T-Antigen
*JCV (J02226.1)*					
21	T	G	0.98	NA	NA
119	T	G	0.99	NA	NA
3135	C (G)	T (A)	0.32	Arg/Lys	Large T-Antigen
4301	G (C)	C (G)	0.39	His/Gln	Large T-Antigen

**Table 3:** Copy number determination using dPCR BKV IS and JCV IS. For BKV, BK-A and BK-B in the VP regions have comparable concentrations, while the assays in the large T-antigen region are reduced by about 75%. Similarly, for JCV, JC-H, JC-I, JC-J show approximately the same concentration, while JC-K and JC-L, located in the large T-antigen regions, are reduced by nearly 80%. JC-M, in the small T-antigen region, has a 43% higher concentration relative to the assays in the VP regions.

Assay	Region	Avg (Copies/mL)	SD (Copies/mL)	Ratio (Relative to BK-A)	Avg (Log_10_)	SD (Log_10_)
BK-A	VP2	3.75 × 10^7^	3.29 × 10^5^	1.00	7.533	0.006
BK-B	VP2	4.13 × 10^7^	9.41 × 10^5^	1.06	7.557	0.008
BK-D	Large T Antigen	4.08 × 10^7^	1.02 × 10^6^	0.24	6.906	0.027
BK-E	Large T Antigen	8.47 × 10^6^	1.35 × 10^5^	0.25	6.930	0.006
BK-F	Large T Antigen	9.00 × 10^6^	2.61 × 10^5^	0.26	6.950	0.020
BK-G	Large T Antigen	5.89 × 10^7^	1.45 × 10^6^	0.27	6.964	0.014
**Assay**	**Region**	**Avg (Copies/mL)**	**SD (Copies/mL)**	**Ratio (Relative to JC-I)**	**Avg (Log_10_)**	**SD (Log_10_)**
JC-H	VP2/VP3	3.42 × 10^7^	4.51 × 10^5^	0.91	7.575	0.004
JC-I	VP1	3.60 × 10^7^	5.24 × 10^5^	1.00	7.616	0.010
JC-J	VP1	8.06 × 10^6^	4.04 × 10^5^	0.99	7.611	0.011
JC-K	Large T Antigen	8.50 × 10^6^	1.02 × 10^5^	0.21	6.928	0.007
JC-L	Large T Antigen	8.92 × 10^6^	3.34 × 10^5^	0.22	6.954	0.013
JC-M	Small T Antigen	9.21 × 10^6^	2.52 × 10^5^	1.43	7.770	0.011

## Data Availability

The data presented in this study are available on request from the corresponding author.
